# Impacts of non-starch polysaccharide sources with enzymes influencing intestinal mucosa-associated microbiota and mucosal immunity of nursery pigs on growth and carcass traits at market weight

**DOI:** 10.1186/s40104-025-01170-4

**Published:** 2025-04-01

**Authors:** Jonathan T. Baker, Zixiao Deng, Alexa R. Gormley, Sung Woo Kim

**Affiliations:** Department of Animal Science, North Carolina State University, 116 Polk Hall, Campus Box 7621, Raleigh, NC 27695 USA

**Keywords:** Intestinal health, Mucosa-associated microbiota, Non-starch polysaccharides, Nursery pigs, Uniformity

## Abstract

**Background:**

This study investigated the effects of different non-starch polysaccharide (NSP) sources with NSP degrading enzymes (NSPases) and the influence on the mucosa-associated microbiota and intestinal immunity of nursery pigs, on growth performance and carcass traits at market weight.

**Methods:**

One hundred and sixty newly weaned pigs at 7.0 ± 0.3 kg body weight (BW) were allotted in a 2 × 2 factorial with NSP sources and NSPases serving as factors. The 4 dietary treatments were: DDGS, corn distillers’ dried grains with solubles as source of NSP; DDGS + NSPases (DDGS +), DDGS with xylanase at 0.01%, 3,000 U/kg of feed and β-mannanase at 0.05%, 400 U/kg of feed; SHWB, soybean hulls and wheat bran replacing corn DDGS as the source of NSP; SHWB with NSPases (SHWB +), SHWB with xylanase at 0.01%, 3,000 U/kg of feed and β-mannanase at 0.05%, 400 U/kg of feed. Pigs were fed for 37 d and housed in groups of 4 pigs per pen. At d 37, the median body weight pig in each pen was euthanized for sampling to analyze intestinal health parameters. Remaining pigs were fed a common diet for subsequent phases to evaluate the carryover effect on growth and carcass traits.

**Results:**

The SHWB decreased (*P* < 0.05) the relative abundance of *Helicobacter*, tended to increase (*P* = 0.074) the relative abundance of *Lactobacillus*, increased (*P* < 0.05) immunoglobulin G (IgG) in the jejunal mucosa, tended to increase (*P* = 0.096) the villus height (VH) in the jejunum, and tended to improve ADG (*P* = 0.099) and feed efficiency (*P* = 0.068) during phase 1 compared to DDGS treatment. Supplementation of NSPases increased (*P* < 0.05) Shannon index of diversity, increased the relative abundance of *Streptococcus* and *Acinetobacter*, and tended to increase (*P* = 0.082) dry matter digestibility. The BW of pigs fed SHWB was more uniform (*P* < 0.05) at the end of the 120 d study. Additionally, hot carcass weight of pigs fed SHWB tended to be more uniform (*P* = 0.089) than DDGS treatment.

**Conclusion:**

Soybean hulls and wheat bran replacing DDGS in nursery diets improved uniformity of pigs at market weight, which might be attributed to beneficial modulation of the mucosa-associated microbiota and enhanced intestinal morphology during the nursery phase. Supplementation of NSPases had beneficial effects on the intestinal mucosa-associated microbiota, digestibility, and intestinal immunity in SHWB treatment, whereas no carryover effects were overserved at market weight.

## Background

Post-weaning nutritional strategies offer a window of opportunity to improve nursery pig health and influence gastrointestinal maturation that could have effects on subsequent performance in later phases of production. A highly diverse community of commensal bacteria within the gut help to prevent potential pathogens, toxins, and other harmful antigens from crossing the epithelium and causing disease [1]. The initial composition of the gut microbiota of pigs is most likely established at birth and then modulated by the sow’s milk through the lactation phase of production, thus altering the microbiota to be characterized by higher abundances of lactic acid bacteria [2]. The composition and diversity of the intestinal microbiota in nursery pigs, however, is highly impacted by the composition of the diet. Dietary fiber plays a crucial role in maintaining a healthy intestinal microbiota by serving as a substrate for microbial fermentation, which leads to the production of short-chain fatty acids (SCFAs). These SCFAs help support intestinal health by promoting the growth of beneficial bacteria, regulating pH levels, and contributing to intestinal barrier function [3].

Characterization of insoluble (IDF) and soluble dietary fiber (SDF) based on the physical properties of solubility have been used to evaluate the roles of dietary fiber more accurately in pig nutrition [4]. In general, IDF is not as easily fermented by intestinal microbiota compared to SDF and stimulates development and peristalsis of the intestine whereas SDF is primarily degraded by intestinal microbiota and may increase digesta viscosity, short-chain fatty acid production, and digesta retention time [5]. Traditionally, high dietary fiber diets have been viewed as negative due to the inherent increase in non-starch polysaccharides (NSP) that cannot be hydrolyzed by endogenous enzymes and anti-nutritional factors associated with the fiber structure that can encapsulate other nutrients, increase endogenous nutrient loss, and result in lower nutrient digestibility [6, 7]. Corn distillers’ dried grains with solubles (DDGS), as a dietary fiber source, has become a major feedstuff in the United States. The excessive DDGS inclusion in nursery diets could impair the growth performance of pigs due to high NSP content [8]. However, some studies report no difference in the digestibility of nutrients when utilizing high-fiber diets, depending on the type of fiber used [9–11], and have been shown to have beneficial effects on gut health and meat quality [12, 13]. For example, it has been indicated that soybean hulls and wheat bran at the appropriate inclusion level can improve performance and fecal microbiota more than other fiber sources [14, 15].

Exogenous carbohydrases such as non-starch polysaccharide degrading enzymes (NSPases) possess the ability to hydrolyze dietary fiber components to release oligosaccharides as well as mitigate the negative effects of dietary fiber on intestinal health, such as increased digesta viscosity [16]. The liberated oligosaccharides can in turn stimulate the proliferation of beneficial bacteria and competitively exclude the growth and colonization of pathogenic bacteria [17, 18]. Previous studies have found beneficial effects on growth performance and nutrient digestibility with NSPases during the nursery phase [19–21]. Some studies have investigated the impacts of different NSP sources on growth, digestibility, intestinal microbiota and health during different phases of production such as nursery, growing-finishing, and sows [22–24], however, there is a gap in knowledge regarding the effects of different NSP sources with NSPases in the nursery diets on subsequent growth performance and carcass traits.

Therefore, it was hypothesized that different NSP sources with NSPases supplementation could affect the intestinal immunity and microbiota of nursery pigs, thereby affecting the growth performance and carcass traits at market weight. The objective of this study was to investigate the effects of different NSP sources with NSPases in the diets and the influence on the mucosa-associated microbiota and intestinal immunity of nursery pigs, on growth performance and carcass traits at market weight.

## Materials and methods

The experimental protocol was approved by the Institutional Animal Care and Use Committee of North Carolina State University (Raleigh, NC, USA). The experiment was conducted at the Central Crops Research Station (Clayton, NC, USA).

### Experimental design, animals, and diets

One hundred and sixty nursery pigs (80 male and 80 female, PIC Camborough × DNA 600) at 7.0 ± 0.3 kg body weight (BW) were allotted in a randomized complete block design in a 2 × 2 factorial arrangement, with NSP source [DDGS vs. soybean hulls and wheat bran (SHWB)] and NSPases (0 vs. 0.06%) as the 2 factors and sex serving as a block. The NSPases used in this study were xylanase at 0.01%, supplying 3,000 U/kg of feed, and β-mannanase at 0.05%, supplying 400 U/kg of feed, for a total of 0.06%, directly replacing corn. Pigs were fed for 37 d in 2 phases: phase 1 from 7 to 11 kg and phase 2 from 11 to 25 kg. Titanium dioxide (0.4%) was added as an indigestible external marker and fed during the last 5 d of phase 2 of the study. All nutrients in the experimental diets met or were slightly higher than the requirement suggested by NRC [25]. All experimental diets (Tables 1 and 2) were produced at the Feed Mill Educational Unit at North Carolina State University (Raleigh, NC, USA). All experimental diets were sampled and sent to the North Carolina Department of Agriculture and Consumer Services (Raleigh, NC, USA) for analysis of nutrient composition. Pigs of the same sex were housed in groups of 4 for the duration of the nursery phase (phase 1 and phase 2: 7 to 25 kg BW). At the end of nursery phase, one pig with the BW closest to the mean BW within a pen (*N* = 40) was selected and euthanized for sample collection to evaluate nutrient digestibility, mucosa-associated microbiota, and oxidative stress and inflammatory indices. The remaining pigs (*N* = 120) were transitioned to a common diet for phase 3 (25 to 50 kg BW), phase 4 (50 to 75 kg BW), phase 5 (75 to 100 kg BW), and phase 6 (100 to 125 kg BW) to evaluate responses to common grow-finish diets after being offered diets with different NSP sources, with or without NSPases, during the nursery phase. At the end of the trial, one finishing pig with the BW closest to the mean BW within a pen (*N* = 40) was selected and euthanized for sample collection to evaluate carcass traits. Total dietary fiber (TDF), SDF, and IDF contents of diets were analyzed based on AOAC Method 991.43 “Total, Soluble, and Insoluble Dietary Fiber in Foods” [26] and AACC Method 32–07.01 [27] using Total Dietary Fiber Assay Kit (Megazyme, Lansing, MI, USA).

**Table 1:** Composition of nursery diets for pigs from 7 to 25 kg BW

Item	P1 (7 kg to 11 kg BW)		P2 (11 kg to 25 kg BW)	
	DDGS^1^	SHWB^1^	DDGS^1^	SHWB^1^
Feedstuff, %				
Corn (yellow)	40.59	36.62	52.12	53.75
Soybean meal (48% CP)	22.00	22.00	25.00	25.00
Whey permeate	13.00	13.00	0.00	0.00
Corn DDGS^2^	10.00	0.00	15.00	0.00
Wheat bran	0.00	6.50	0.00	6.50
Soybean hulls	0.00	6.50	0.00	6.80
Fish meal	3.00	3.00	0.00	0.00
Poultry meal	3.00	3.00	0.00	0.00
Blood plasma	2.00	2.00	0.00	0.00
Poultry fat	4.20	5.00	4.80	4.50
L-Lys HCl	0.41	0.41	0.51	0.53
DL-Met	0.13	0.15	0.12	0.16
L-Thr	0.09	0.13	0.12	0.18
L-Ile	0.00	0.05	0.00	0.08
L-Trp	0.00	0.00	0.00	0.01
L-Val	0.00	0.06	0.00	0.08
L-Iso	0.00	0.05	0.00	0.08
Dicalcium phosphate	0.20	0.30	0.75	1.05
Limestone	0.95	0.80	1.15	0.85
Vitamin premix^3^	0.03	0.03	0.03	0.03
Trace mineral premix^4^	0.15	0.15	0.15	0.15
Salt	0.25	0.25	0.25	0.25
Total	100.00	100.00	100.00	100.00
Calculated composition				
DM, %	90.48	90.58	89.77	89.73
ME, kcal/kg	3,402	3,403	3,350	3,353
CP, %	22.98	21.57	21.17	18.92
SID^5^ Lys, %	1.35	1.35	1.23	1.23
Ca, %	0.80	0.80	0.70	0.70
STTD P^6^, %	0.40	0.40	0.33	0.33
Analyzed composition				
DM, %	88.48	88.62	87.20	87.28
CP, %	24.04	21.99	21.14	18.62
NDF, %	7.81	10.65	9.50	10.80
ADF, %	3.52	5.28	4.68	6.05
IDF^7^, %	10.57	13.63	11.25	16.35
SDF^8^, %	0.58	1.01	0.68	1.25
TDF^9^, %	11.12	14.63	11.92	17.60

^1^Xylanase at 0.01% (3,000 U/kg of feed) and β-mannanase at 0.05% (400 U/kg of feed) for a total of 0.06% NSPases directly replacing corn

^2^ *DDGS* Distillers dried grains with solubles

^3^The vitamin premix provided per kilogram of complete diet: 6,614 IU of vitamin A as vitamin A acetate, 992 IU of vitamin D_3_, 19.8 IU of vitamin E, 2.64 mg of vitamin K as menadione sodium bisulfate, 0.03 mg of vitamin B_12_, 4.63 mg of riboflavin, 18.52 mg of D-pantothenic acid as calcium panthonate, 24.96 mg of niacin, and 0.07 mg of biotin

^4^The trace mineral premix provided per kilogram of complete diet: 33 mg of Mn as manganous oxide, 110 mg of Fe as ferrous sulfate, 110 mg of Zn as zinc sulfate, 16.5 mg of Cu as copper sulfate, 0.30 mg of I as ethylenediamine dihydroiodide, and 0.30 mg of Se as sodium selenite

^5^ *SID* Standardized ileal digestible

^6^ *STTD P* Standardized total tract digestible phosphorus

^7^ *IDF* Insoluble dietary fiber

^8^ *SDF* Soluble dietary fiber

^9^ *TDF* Total dietary fiber. Calculated as the sum of IDF and SDF

**Table 2:** Composition of common diets for growing and finishing pigs from 25 to 125 kg BW

Item	P3 (25 to 50 kg)	P4 (50 to 75 kg)	P5 (75 to 100 kg)	P6 (100 to 125 kg)
Feedstuff, %				
Corn (yellow)	59.90	61.36	66.39	64.61
Corn DDGS^1^	20.00	21.00	21.00	21.00
Soybean meal	12.00	10.00	5.70	7.60
Poultry fat	4.90	5.00	4.50	4.50
L-Lys HCl	0.58	0.47	0.45	0.39
DL-Met	0.09	0.03	0.00	0.00
L-Thr	0.13	0.08	0.08	0.05
L-Trp	0.09	0.03	0.03	0.02
Dicalcium phosphate	0.68	0.40	0.30	0.28
Limestone	1.20	1.20	1.12	1.12
Vitamin premix^2^	0.03	0.03	0.03	0.03
Trace mineral premix^3^	0.15	0.15	0.15	0.15
Salt	0.25	0.25	0.25	0.25
Total	100.00	100.00	100.00	100.00
Calculated composition				
DM, %	89.57	89.50	89.39	89.41
ME, kcal/kg	3,300	3,302	3,298	3,296
CP, %	16.99	16.19	14.70	15.37
SID^4^ Lys, %	0.98	0.85	0.73	0.73
Ca, %	0.66	0.59	0.52	0.52
STTD P^5^, %	0.31	0.27	0.24	0.24
Analyzed composition				
DM, %	87.77	88.09	86.93	86.06
CP, %	19.42	18.46	15.25	16.14
NDF, %	12.10	12.50	12.82	13.98
ADF, %	4.52	4.70	5.65	6.30
IDF^6^, %	12.59	13.58	13.81	14.12
SDF^7^, %	0.44	0.52	0.41	0.56
TDF^8^, %	13.03	14.10	14.22	14.68
CF, %	9.42	9.34	7.77	7.78

^1^ *DDGS* Distillers dried grains with solubles

^2^The vitamin premix provided per kilogram of complete diet: 6,614 IU of vitamin A as vitamin A acetate, 992 IU of vitamin D_3_, 19.8 IU of vitamin E, 2.64 mg of vitamin K as menadione sodium bisulfate, 0.03 mg of vitamin B_12_, 4.63 mg of riboflavin, 18.52 mg of D-pantothenic acid as calcium panthonate, 24.96 mg of niacin, and 0.07 mg of biotin

^3^The trace mineral premix provided per kilogram of complete diet: 33 mg of Mn as manganous oxide, 110 mg of Fe as ferrous sulfate, 110 mg of Zn as zinc sulfate, 16.5 mg of Cu as copper sulfate, 0.30 mg of I as ethylenediamine dihydroiodide, and 0.30 mg of Se as sodium selenite

^4^ *SID* Standardized ileal digestible

^5^ *STTD P* Standardized total tract digestible phosphorus

^6^ *IDF* Insoluble dietary fiber

^7^ *SDF* Soluble dietary fiber

^8^ *TDF* Total dietary fiber. Calculated as the sum of IDF and SDF

### Experimental procedures and sample collection

The BW of each pig within a pen was measured and recorded every 7 d during the nursery phase to calculate average daily gain (ADG), and gain:feed (G:F). Average daily feed intake (ADFI) was measured on a pen basis using feed disappearance divided by the number of days and pigs within a pen. In the grow-finish phases (phase 3 to phase 6), pigs were weighed per 2 weeks with growth performance being measured and calculated in the same way as the nursery phase. At the end of the nursery phase (d 37), one pig with the BW closest to the mean BW within each pen was euthanized by a captive bolt gun followed by exsanguination and removal of the gastrointestinal tract for sample collection. Ileal digesta was collected in a 100-mL container and put on the ice, then stored at −20 °C for measurement of apparent ileal digestibility (AID) of nutrients. Jejunal digesta was collected into 50-mL falcon tube and placed on ice then immediately transferred to the lab for measurement of digesta viscosity. Mid-jejunum segments were rinsed with 0.9% saline solution and collected in a 50-mL falcon tube with 10% buffered formaldehyde to evaluate histology. Mucosal samples from mid-jejunum were scraped by a glass slide and collected in Eppendorf tubes (2 mL), then put it into liquid nitrogen immediately and stored at −80 °C for subsequent immune, oxidative stress, and mucosa-associated microbiota measurements.

### Digesta viscosity

Following the procedure by Passos et al. [28] and Duarte et al. [29], samples of jejunal digesta from 50-mL tubes were divided into 2 falcon tubes (15 mL) and centrifuged at 1,000 × *g* at 4 °C for 10 min to obtain the liquid phase. The liquid phase was then removed and transferred to an Eppendorf tube (2 mL) to centrifuge at 10,000 × *g* at 4 °C for 10 min. The supernatant obtained was transferred to another Eppendorf tube (1.5 mL) for further measurement. A total of 0.5 mL of digesta supernatant was placed in the viscometer (Brookfield Digital Viscometer, Model DV-II Version 2.0, Brookfield Engineering Laboratories Inc., Stoughton, MA, USA), set at 25 °C. The viscosity measurement was reported as the average between 45.0 s^−1^ and 22.5 s^−1^ shear rates, and the viscosity values were recorded as apparent viscosity in centipoise (cP).

### Relative abundance and diversity of jejunal mucosa-associated microbiota

Mid-jejunum mucosa samples were sent to Zymo Research for DNA extraction and microbiota sequencing according to Zymo Research internal protocols. In short, DNA was extracted by Zymo Research (Irvine, CA, USA) using ZymoBIOMICS-96 MagBead DNA Kit (Zymo Research, Irvine, CA, USA). The DNA samples were prepared for targeted sequencing with the Quick-16S Plus NGS Library Prep Kit (Zymo Research, Irvine, CA, USA) and the primer set used was Quick-16S Primer Set V3–V4 (Zymo Research, Irvine, CA, USA). The final PCR products were quantified with qPCR fluorescence readings and pooled together based on equal molarity. The final pooled library was cleaned up with the Select-a-Size DNA Clean & Concentrator (Zymo Research, Irvine, CA, USA), then quantified with TapeStation (Agilent Technologies, Santa Clara, CA, USA) and Qubit (Thermo Fisher Scientific, Waltham, WA, USA). The final library was sequenced on Illumina NextSeq 2000 with a p1 (Cat. 20075294) reagent kit (600 cycles). The sequencing was performed with 30% PhiX spike-in. Unique amplicon sequences were inferred from raw reads using the Dada2 pipeline [30]. Chimeric sequences were also removed with the DADA2 pipeline. Taxonomy was assigned based on Greengenes and Silva. To initiate the statistical analysis of the microbiota, ASV data were transformed to relative abundance as previously described by Kim et al. [31]. The ASV with the relative abundance < 0.5% within each level were combined as “Others”.

### Inflammatory cytokines, immunoglobulins, and oxidative damage products

Jejunal mucosa samples were weighed (1 g) and suspended in 1 mL of phosphate-buffered saline (PBS) on ice, then homogenized using a tissue homogenizer (Tissuemiser; Thermo Fisher Scientific Inc., Waltham, MA, USA). Following Holanda and Kim [32], the processed samples were then transferred into a new 2-mL microcentrifuge tube and centrifuged at 14,000 × *g* for 15 min. The supernatant was pipetted into 5 aliquots and stored at −80 °C. The concentration of total protein, interleukin 6 (IL-6), and interleukin 8 (IL-8), tumor necrosis factor-alpha (TNF-α), immunoglobulin G (IgG), immunoglobulin A (IgA), malondialdehyde (MDA), and protein carbonyl (PC) were measured by using commercial kits based on the instruction manual. The OD value was read by the ELISA plate reader (Synergy HT, BioTek Instruments, Winooski, VT, USA) and software (Gen5 Data Analysis Software, BioTek Instruments). The corresponding concentrations were calculated according to the absorbance of standard curves and instruction manual. The homogenized mucosal supernatant was diluted (1:60) in PBS to get the appropriate range (20–2000 μg/mL), then the total protein concentration was measured by using Pierce BCA Protein Assay Kit (#23225, Thermo Fisher Scientific Inc.) as described by Holanda et al. [33]. The absorbance was measured at 562 nm and the concentration of total protein were further used to normalize the concentration of other measurements in mucosa. The concentration of IL-6 in jejunal mucosa was measured by following instructions of the Porcine IL-6 DuoSet ELISA Kit (DY686, R&D Systems, Minneapolis, MN, USA) as described by Duarte et al. [34]. The concentration of IL-6 was described as pg/mg of protein. The concentration of IL-8 was measured by using Porcine IL-8/CXCL8 DuoSet ELISA kit (#DY535, R&D Systems) as described by Jang and Kim [35]. All sample were diluted in reagent diluent to 1:5 to analyze. Absorbance was read at 450 nm and corrected at 570 nm. The concentration was expressed as pg/mL protein. TNF-α concentration was measured by using Porcine TNF-α Immunoassay Kit (#PTA00, R&D Systems, Minneapolis, MN, USA) as described by Cheng et al. [36]. Absorbance was read at 450 nm and corrected at 570 nm. The concentration of TNF-α was expressed as pg/mL protein. The concentration of IgA and IgG was measured by using the ELISA kits (E101-102 and E101-104, Bethyl Laboratories, Inc., Montgomery, TX, USA) as described by Holanda et al. [33]. The mucosal supernatants were diluted with PBS to 1:1,200 and 1:2,400, respectively, to get the appropriate working range for measurement. Absorbance was read at 450 nm and the concentration was expressed as μg/mg of protein. The concentration of MDA in mucosa was measured by using OxiSelect TBARS MDA Quantitation Assay Kit (#STA-330, Cell Biolabs, Inc.) as described by Moita et al. [37]. The working range of standard is from 0.98 to 125 µmol/L. The absorbance was read under 532 nm wavelength. The concentration was calculated according to standard and expressed as µmol/mg protein. Protein carbonyl was measured by using OxiSelect Protein Carbonyl ELISA Kit (#STA-310, Cell Biolabs, Inc., San Diego, CA, USA) as described by Moita et al. [37]. All supernatants were diluted in PBS to get 10 µg/mL before measurement. The standard was prepared that range was from 0.375 to 7.5 nmol/mg protein. All processes conducted following the manufacturer’s protocol. The absorbance was measured at 450 nm and the concentration was described as nmol/mg protein.

### Intestinal morphology and Ki-67 in crypt cells

Two sections of mid jejunum per pig were fixed in 10% formalin and then were transferred to a 70% ethanol solution for 2 d. The processed samples were sent to North Carolina State University Histology Laboratory (College of Veterinary Medicine, Raleigh, NC, USA) for dehydration, embedment, staining and Ki-67 assay. Villus height (VH) and crypt depth (CD) were measured using a microscope Olympus CX31 (Lumenera Corporation, Ottawa, CA) with a software of Infinity 2–2 digital CCD. In each slide, 10 intact villi and their associated crypts were measured as described by Cheng et al. [36]. The villus length was measured from the top of the villus to the junction of villus and crypt; the villus width was measured in the middle of the villus; the crypt depth was measured from the junction of villus and crypt to the bottom of the crypt. The villus height to crypt depth (VH:CD) ratio was calculated using the villus height divided by the crypt depth. Images of 10 intact crypts from each slide were cropped, and the ImageJS software was used for calculating the percentage of Ki-67 positive cells to total cells in the crypt. All analyses of the intestinal morphology were executed by the same person. The averages of the 10 measurements per pig were calculated and reported as one number per pig.

### Apparent ileal digestibility

Titanium dioxide was added at 0.4% to phase 3 diets to serve as an indigestible marker to determine the apparent ileal digestibility (AID) of nutrients. Ileal digesta were freeze-dried for 48 h (24D 48, Virtis, Gardiner, NY, USA). The concentration of titanium dioxide in the feed and digesta were measured based on the approach of Myers et al. [38]. The feed and digesta samples were used to measure the content of dry matter (DM, method 934.01) and ether extract (EE, method 2003.06) based on AOAC [39]. Gross energy (GE) was measured using a bomb calorimeter (Model 6200, Parr Instrument Company, Moline, IL, USA). The nitrogen content was measure using TruSpec N Nitrogen Determinator (LECO CN-2000, LECO Corp., St. Joseph, MI) and the CP concentration was calculated (6.25 × N). The AID of DM, GE, EE, and CP were calculated by using following function:

$$\text{AID }= \{1- [({\text{TiO}}_{2\text{feed}} /{\text{ TiO}}_{2\text{digesta}}) \times ({\text{Nutrient}}_{\text{digesta}}/{\text{Nutrient}}_{\text{feed}})]\} \times 100$$

In which TiO_2feed_ and TiO_2digesta_ were the measured concentration of titanium dioxide in the feed and in the digesta; Nutrient_digesta_ and Nutrient_feed_ were the measured concentration of nutrient in the digesta and in the feed as previously described by Moita et al. [37].

### Carcass traits

The day prior to slaughter, the pigs were weighed to determine their final body weight. They were then transported to a local processing plant, where they were humanely stunned using electrical methods and processed in accordance with industry standards. The hot carcass weight was weighed immediately. Dressing percentage was calculated using the following equation and measurements obtained during processing:

$$\mathrm{Dressing}\;\mathrm{percentage}\ \left(\%\right)=\left(\mathrm{Carcass}\;\mathrm{weight}/\mathrm{Slaughter}\;\mathrm{weight}\right)\times100$$

Loin eye area and loin depth, and backfat depth measurements were taken between the 10^th^ and 11^th^ ribs, and at the 10^th^ rib, on the left half of the carcass, respectively, using machinery available at the processing plant. Percentage of lean was calculated using the following equation [40] and measurements obtained during processing:

$$\mathrm{Lean}\ \left(\%\right)=\left[\left(8.588-21.896\times\mathrm{backfat}\;\mathrm{depth}\left(\mathrm{inches}\right)+3.005\times\mathrm{loin}\;\mathrm{eyes}\;\mathrm{area}\left(\mathrm{square}\;\mathrm{inches}\right)+0.465\times\mathrm{hot}\;\mathrm{carcass}\;\mathrm{weight}\right)/\mathrm{hot}\;\mathrm{carcass}\;\mathrm{weight}\right]\times100\\$$

### Statistical analysis

Data were analyzed based on a randomized complete block design using the MIXED model of SAS 9.4 (Cary, NC, USA). Experimental unit was the pen. Factors and their interactions were evaluated as fixed effects and sex blocks served as random effects. Homogeneity of variance BW and hot carcass weight were tested using Levene’s test of the MIXED procedure, which revealed that the variance of residuals of several dependent variables was unequal. Statistical differences will be considered significant with *P* < 0.05 and tendency with 0.05 ≤ *P* < 0.10. The microbiome data were tested for normal distribution with the UNIVARIATE (Shapiro–Wilk test), and the non-normally distributed data were analyzed using the GLIMMIX procedure through Poisson distributions according to Zhang et al. [41].

## Results

### Digesta viscosity

**Fig. 1:**
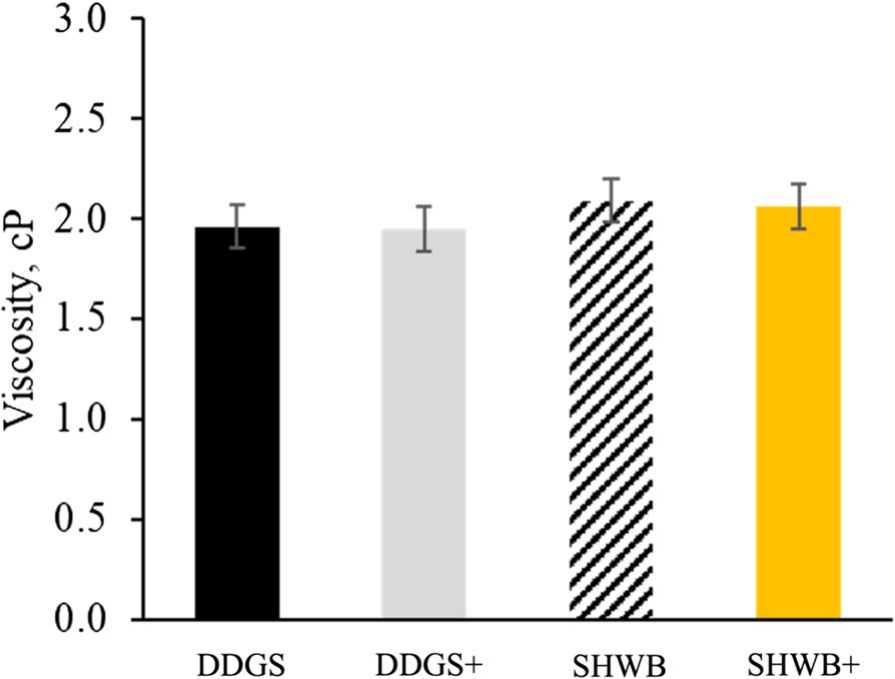
Changes in the viscosity of jejunal digesta in nursery pigs fed diets with different NSP source and NSPases. DDGS: 10% corn DDGS in 7 to 11 kg diets and 15% corn DDGS in 11 to 25 kg diets; DDGS+: 10% corn DDGS in 7 to 11 kg diets and 15% corn DDGS in 11 to 25 kg diets + xylanase at 0.01% (3,000 U/kg of feed) and β-mannanase at 0.05% (400 U/kg of feed); SHWB: 6.5% wheat bran and soybean hulls in 7 to 11 kg diets and 6.8% wheat bran and soybean hulls in 11 to 25 kg diets; SHWB+: 6.5% wheat bran and soybean hulls in 7 to 11 kg diets and 6.8% wheat bran and soybean hulls in 11 to 25 kg diets + xylanase at 0.01% (3,000 U/kg of feed) and β-mannanase at 0.05% (400 U/kg of feed)

The jejunal viscosity of nursery pigs was not affected by NSP sources or NSPases (Fig. 1).

### Diversity and relative abundance of jejunal mucosa-associated microbiota

Different NSP sources nor NSPases had an effect on Chao1 α-diversity of jejunal mucosa-associated microbiota at the species level however, NSPases increased (*P* < 0.05) the Shannon index (Table 3). At the phylum level, SHWB decreased (*P* < 0.05) the relative abundance of Proteobacteria and increased (*P* < 0.05) Actinobacteria whereas NSPases decreased (*P* < 0.05) the relative abundance of Tenericutes and Bacteroidetes (Table 4). Additionally, supplementation of NSPases in SHWB increased (*P* < 0.05) the relative abundance of Firmicutes, decreased Actinobacteria (*P* < 0.05), and tended to increase (*P* = 0.096) Bacteroidetes.

**Table 3:** α-Diversity of jejunal mucosa-associated microbiota at the Species level estimated with Chao1 richness, Shannon diversity, and Simpson diversity in nursery pigs fed different NSP sources with or without NSPases

NSP type^1^	DDGS		SHWB		SEM	*P* value		
NSPases^2^	-	+	-	+		NSP	NSPases	NSP × NSPases
Chao1	263.2	374.6	233.0	308.9	67.1	0.482	0.175	0.794
Shannon	4.5	5.2	4.3	5.1	0.3	0.751	0.032	0.842
Simpson	0.9	0.9	0.9	0.9	0.1	0.888	0.149	0.455

*N* = 40 for total, *n* = 10 for each treatment

^1^The DDGS included 10% corn DDGS in 7 to 11 kg diets and 15% corn DDGS in 11 to 25 kg diets. The SHWB included 6.5% wheat bran and soybean hulls in 7 to 11 kg diets and 6.8% wheat bran and soybean hulls in 11 to 25 kg diets

^2^NSPases containing 0.01% xylanase and 0.05% beta-mannanase was supplemented into experimental diets at 0.06%

**Table 4:** Relative abundance of jejunal mucosa-associated microbiota at the Phylum level in nursery pigs fed different NSP sources with or without NSPases

NSP type^1^	DDGS		SHWB		SEM	*P* value		
NSPases^2^	-	+	-	+		NSP	NSPases	NSP × NSPases
Firmicutes	63.30^ab^	59.20^ab^	57.12^b^	65.90^a^	5.41	0.961	0.422	0.033
Actinobacteria	10.18^a^	14.02^a^	21.43^b^	13.72^a^	2.18	0.002	0.546	0.001
Proteobacteria	14.90	12.86	3.84	4.83	3.02	< 0.001	0.772	0.236
Tenericutes	1.26	0.01	1.17	0.00	1.65	0.693	0.001	0.737
Bacteroidetes	1.02^ab^	2.75^ac^	0.30^b^	3.44^c^	0.52	0.243	< 0.001	0.096
Others	1.19	1.54	0.70	1.50	0.40	0.437	0.158	0.478

*N* = 40 for total, *n* = 10 for each treatment

^1^The DDGS included 10% corn DDGS in 7 to 11 kg diets and 15% corn DDGS in 11 to 25 kg diets. The SHWB included 6.5% wheat bran and soybean hulls in 7 to 11 kg diets and 6.8% wheat bran and soybean hulls in 11 to 25 kg diets

^2^NSPases containing 0.01% xylanase and 0.05% beta-mannanase was supplemented into experimental diets at 0.06%

^a,b,c^Within a row, means lacking a common superscript letter differ (*P* < 0.05)

At the family level, SHWB tended to increase (*P* = 0.074) the relative abundance of Lactobacillaceae, increased (*P* < 0.05) Bifidobacteriaceae, and decreased (*P* < 0.05) the relative abundance of Streptococcaceae and Helicobacteraceae (Table 5). The NSPases decreased (*P* < 0.05) the relative abundance of Lactobacillaceae and bacteria labeled as Other, and increased (*P* < 0.05) the relative abundance of Streptococcaceae, Lachnospiraceae, Veillonellaceae, and Prevotellaceae. Moreover, supplementation of NSPases in SHWB increased (*P* < 0.05) Streptococcaceae and decreased (*P* < 0.05) Bifidobacteriaceae and Erysipelotrichaceae.

**Table 5:** Relative abundance of jejunal mucosa-associated microbiota at the Family level in nursery pigs fed different NSP sources with or without NSPases

NSP type^1^	DDGS		SHWB		SEM	*P* value		
NSPases^2^	-	+	-	+		NSP	NSPases	NSP × NSPases
Lactobacillaceae	37.41	27.57	37.37	34.84	2.65	0.075	0.006	0.073
Streptococcaceae	14.53^a^	15.80^a^	7.10^b^	16.16^a^	1.69	0.003	< 0.001	0.002
Bifidobacteriaceae	9.14^a^	12.45^a^	20.07^b^	12.45^a^	2.11	0.002	0.443	0.002
Helicobacteraceae	12.92	9.56	3.17	3.89	3.70	< 0.001	0.756	0.129
Lachnospiraceae	3.84	5.81	5.03	6.39	1.81	0.257	0.047	0.585
Veillonellaceae	2.70	3.18	1.16	2.92	0.57	0.080	0.042	0.149
Ruminococcaceae	1.29	2.62	2.08	2.31	0.78	0.505	0.124	0.254
Erysipelotrichaceae	1.05^a^	1.41^ab^	3.08^b^	1.08^a^	0.46	0.206	0.239	0.040
Prevotellaceae	0.87	2.12	0.18	2.63	0.46	0.203	0.002	0.097
Coriobacteriaceae	0.75	1.10	1.22	0.84	0.36	0.776	0.988	0.331
Other	10.96	7.25	8.95	6.31	5.34	0.174	0.004	0.796

*N* = 40 for total, *n* = 10 for each treatment

^1^The DDGS included 10% corn DDGS in 7 to 11 kg diets and 15% corn DDGS in 11 to 25 kg diets. The SHWB included 6.5% wheat bran and soybean hulls in 7 to 11 kg diets and 6.8% wheat bran and soybean hulls in 11 to 25 kg diets

^2^NSPases containing 0.01% xylanase and 0.05% beta-mannanase was supplemented into experimental diets at 0.06%

^a,b^Within a row, means lacking a common superscript letter differ (*P* < 0.05)

At the genus level, SHWB increased (*P* < 0.05) the relative abundance of *Bifidobacterium* and tended to increase (*P* = 0.074) the relative abundance of *Lactobacillus* (Table 6). Moreover, SHWB decreased (*P* < 0.05) the relative abundance of *Streptococcus*, *Helicobacter*, and tended to decrease (*P* = 0.081) *Megasphaera*. The NSPases decreased (*P* < 0.05) the relative abundance of *Lactobacillus* and increased (*P* < 0.05) the relative abundance of *Streptococcus* and *Acinetobacter*. Additionally, supplementation of NSPases in SHWB increased (*P* < 0.05) *Streptococcus* and decreased (*P* < 0.05) *Bifidobacterium*.

**Table 6:** Relative abundance of jejunal mucosa-associated microbiota at the Genus level in nursery pigs fed different NSP sources with or without NSPases

NSP type^1^	DDGS		SHWB		SEM	*P* value		
NSPases^2^	-	+	-	+		NSP	NSPases	NSP × NSPases
*Lactobacillus*	37.39	27.55	37.37	34.82	2.65	0.074	0.006	0.073
*Streptococcus*	14.53^a^	15.80^a^	7.10^b^	16.16^a^	1.69	0.003	< 0.001	0.002
*Bifidobacterium*	9.14^a^	12.45^a^	20.07^b^	12.45^a^	2.11	0.002	0.443	0.002
*Helicobacter*	12.92	9.56	3.17	3.89	3.70	< 0.001	0.756	0.129
*Acetitomaculum*	1.31	1.51	1.97	2.31	0.91	0.126	0.574	0.979
*Megasphaera*	1.37	1.62	0.52	1.08	0.38	0.081	0.244	0.458
*Acinetobacter*	0.10	0.69	0.36	0.53	0.63	0.370	0.047	0.172
*Olsenella*	0.45	0.79	0.92	0.60	0.30	0.637	0.883	0.281
Other	19.73	19.93	19.03	18.49	4.20	0.515	0.914	0.819

*N* = 40 for total, *n* = 10 for each treatment

^1^The DDGS included 10% corn DDGS in 7 to 11 kg diets and 15% corn DDGS in 11 to 25 kg diets. The SHWB included 6.5% wheat bran and soybean hulls in 7 to 11 kg diets and 6.8% wheat bran and soybean hulls in 11 to 25 kg diets

^2^NSPases containing 0.01% xylanase and 0.05% beta-mannanase was supplemented into experimental diets at 0.06%

^a,b^Within a row, means lacking a common superscript letter differ (*P* < 0.05)

At the species level, SHWB increased (*P* < 0.05) the relative abundance of *Bifidobacterium dentium, Lactobacillus* sp29233*, Lactobacillus equicursoris, Lactobacillus delbrueckii-*sp29223*, Lactobacillus salivarius,* and *Lactobacillus johnsonii,* and tended to increase (*P* = 0.081) the relative abundance of *Acetitomaculum* sp31898 (Table 7). Additionally, SHWB decreased (*P* < 0.05) the relative abundance of *Helicobacter ganmani, Streptococcus alactolyticus,* and *Lactobacillus* sp.*,* and tended to decrease (*P* = 0.071) the relative abundance of *Lactobacillus fermentum.* The NSPases increased (*P* < 0.05) the relative abundance of *Lactobacillus delbrueckii, Streptococcus* sp.*, Streptococcus alactolyticus,* bacteria labeled as Other, and tended to increase (*P* = 0.074) *Helicobacter equorum* and *Helicobacter ganmani* (*P* = 0.090). Moreover, NSPases decreased the relative abundance of *Lactobacillus* sp29233 and *Lactobacillus delbrueckii-*sp29223 and tended to decrease (*P* = 0.065) *Bifidobacterium boum.* Additionally, supplementation of NSPases in SHWB increased (*P* < 0.05) *Bifidobacterium dentium* and decreased (*P* < 0.05) *Helicobacter ganmani* in DDGS diets whereas decreased (*P* < 0.05, interaction) *Bifidobacterium dentium*, increased (*P* < 0.05) *Helicobacter ganmani*, and increased (*P* < 0.05) *Streptococcus alactolyticus*.

**Table 7:** Relative abundance of jejunal mucosa-associated microbiota at the Species level in nursery pigs fed different NSP sources with or without NSPases

NSP type^1^	DDGS		SHWB		SEM	*P* value		
NSPases^2^	-	+	-	+		NSP	NSPases	NSP × NSPases
*Lactobacillus delbrueckii*	9.88	11.17	9.27	13.38	1.86	0.591	0.030	0.262
*Bifidobacterium dentium*	6.36^a^	10.39^b^	15.69^c^	10.55^b^	1.27	0.001	0.716	0.001
*Streptococcus* sp.	6.40	9.14	4.37	10.75	2.05	0.418	< 0.001	0.050
*Helicobacter rappini*	5.33	7.91	8.06	8.19	1.26	0.101	0.128	0.159
*Lactobacillus* sp.	8.11	6.62	2.29	3.31	2.64	< 0.001	0.632	0.106
*Helicobacter ganmani*	7.61^a^	4.10^b^	1.96^b^	6.84^a^	0.78	0.026	0.090	< 0.001
*Lactobacillus mucosae*	4.13	3.58	4.41	4.14	0.89	0.551	0.558	0.819
*Streptococcus alactolyticus*	5.37^a^	5.70^a^	1.18^b^	3.88^a^	1.14	< 0.001	0.007	0.013
*Lactobacillus sp29233*	2.43	0.86	6.53	2.22	0.99	0.001	< 0.001	0.943
*Lactobacillus equicursoris*	1.15^a^	1.84^a^	4.76^b^	2.58^ab^	1.01	0.001	0.766	0.032
*Helicobacter equorum*	0.02	4.28	0.00	0.07	1.66	0.218	0.074	0.578
*Acetitomaculum sp31898*	1.11	1.51	1.94	2.25	0.85	0.081	0.391	0.755
*Lactobacillus delbrueckii-sp29223*	1.53	0.41	3.71	1.34	0.59	0.007	0.003	0.682
*Bifidobacterium thermacidophilum-thermophilum*	1.13	1.18	2.78	1.33	0.63	0.120	0.286	0.233
*Bifidobacterium boum*	1.57	1.09	1.63	0.57	0.65	0.412	0.065	0.360
*Megasphaera sp36946*	1.21	1.60	0.55	1.07	0.37	0.114	0.204	0.596
*Lactobacillus reuteri-vaginalis*	1.36^a^	0.44^ab^	0.35^b^	1.52^a^	0.54	0.912	0.708	0.005
*Lactobacillus salivarius*	0.17	0.27	0.74	1.12	0.31	0.015	0.386	0.937
*Lactobacillus johnsonii*	0.18	0.07	0.34	1.65	0.38	0.035	0.695	0.149
*Lactobacillus fermentum*	1.26	0.30	0.20	0.21	0.46	0.071	0.254	0.228
Other	13.70	22.16	13.55	23.00	1.49	0.879	< 0.001	0.782

^1^The DDGS included 10% corn DDGS in 7 to 11 kg diets and 15% corn DDGS in 11 to 25 kg diets. The SHWB included 6.5% wheat bran and soybean hulls in 7 to 11 kg diets and 6.8% wheat bran and soybean hulls in 11 to 25 kg diets

^2^NSPases containing 0.01% xylanase and 0.05% beta-mannanase was supplemented into experimental diets at 0.06%

^a,b,c^Within a row, means lacking a common superscript letter differ (*P* < 0.05)

### Intestinal inflammatory status, humoral immune status, and oxidative stress status

Concentrations of pro-inflammatory cytokines, IL-6, IL-8, and TNF-α were unaffected by increased NSP source or NSPases. The supplementation of NSPases in SHWB did however, tend to decrease (*P* = 0.053) IL-6 in pigs, whereas no effect was seen in the DDGS diets (Table 8). The concentration of IgG in the mucosa of the jejunum was higher (*P* < 0.05) in the SHWB diets, however concentrations of IgA were unaffected. Moreover, supplementation of NSPases in DDGS increased (*P* < 0.05) the mucosal concentrations of oxidative stress product, protein carbonyl, whereas had no effect in SHWB.

**Table 8:** Oxidative stress and immune parameters in nursery pigs fed different NSP sources with or without NSPases

NSP type^1^	DDGS		SHWB		SEM	*P* value		
NSPases^2^	-	+	-	+		NSP	NSPases	NSP × NSPases
IL-6^3^, pg/mg	16.08^ab^	20.19^a^	20.05^a^	15.29^b^	2.37	0.834	0.885	0.053
IL-8, ng/mg	0.64	0.51	0.62	0.62	0.09	0.580	0.399	0.412
TNF-α^4^, pg/mg	0.77	0.69	0.88	0.88	0.10	0.144	0.677	0.702
IgG^5^, µg/mg	5.14	4.53	5.91	5.74	0.35	0.012	0.302	0.563
IgA, µg/mg	8.79	8.32	7.76	6.58	0.90	0.143	0.375	0.698
MDA^6^, µmol/mg	0.57	0.41	0.58	0.61	0.08	0.224	0.430	0.272
PC^7^, nmol/mg	2.31^a^	2.62^b^	2.55^ab^	2.34^a^	0.11	0.882	0.666	0.031

*N* = 40 for total, *n* = 10 for each treatment

^1^The DDGS included 10% corn DDGS in 7 to 11 kg diets and 15% corn DDGS in 11 to 25 kg diets. The SHWB included 6.5% wheat bran and soybean hulls in 7 to 11 kg diets and 6.8% wheat bran and soybean hulls in 11 to 25 kg diets

^2^NSPases containing 0.01% xylanase and 0.05% beta-mannanase was supplemented into experimental diets at 0.06%

^3^ *IL* Interleukin

^4^ *TNF-α* Tumor necrosis factor α

^5^ *Ig* Immunoglobulin

^6^ *MDA* Malondialdehyde

^7^ *PC* Protein carbonyl

^a,b^Within a row, means lacking a common superscript letter differ (*P* < 0.05)

### Intestinal morphology and crypt cell proliferation

The VH tended to be increased (*P* = 0.096) in the SHWB treatment compared to DDGS with CD, VH:CD, and Ki67^+^ being unaffected among the treatments (Table 9).

**Table 9:** Intestinal morphology and crypt cell proliferation in nursery pigs fed different NSP sources with or without NSPases

NSP type^1^	DDGS		SHWB		SEM	*P* value		
NSPases^2^	-	+	-	+		NSP	NSPases	NSP × NSPases
VH^3^	476.4	489.1	506.1	507.1	13.9	0.096	0.626	0.677
CD^4^	258.3	258.7	260.4	264.4	7.3	0.596	0.765	0.809
VH:CD	1.85	1.90	1.94	1.94	0.06	0.235	0.690	0.636
Ki67^+5^	25.86	24.69	24.06	24.91	1.06	0.459	0.878	0.347

*N* = 40 for total, *n* = 10 for each treatment

^1^The DDGS included 10% corn DDGS in 7 to 11 kg diets and 15% corn DDGS in 11 to 25 kg diets. The SHWB included 6.5% wheat bran and soybean hulls in 7 to 11 kg diets and 6.8% wheat bran and soybean hulls in 11 to 25 kg diets

^2^NSPases containing 0.01% xylanase and 0.05% beta-mannanase was supplemented into experimental diets at 0.06%

^3^ *VH* Villus height

^4^ *CD* Crypt depth

^5^Ratio of Ki-67 positive cells to total cells in the crypt

### Apparent ileal digestibility of nutrients

Supplementation of NSPases tended to increase (*P* = 0.082) AID of DM whereas, dietary NSP tended to decrease (*P* = 0.085) AID of EE (Table 10). The apparent ileal digestibility of GE and CP were not affected by NSP source or NSPases.

**Table 10:** Apparent ileal digestibility of nutrients in nursery pigs fed different NSP sources with or without NSPases

NSP type^1^	DDGS		SHWB		SEM	*P* value		
NSPases^2^	-	+	-	+		NSP	NSPases	NSP × NSPases
Dry matter	57.3	60.1	55.1	58.5	2.8	0.281	0.082	0.842
Gross energy	52.4	55.0	49.7	56.0	3.4	0.762	0.114	0.506
Crude protein	58.8	61.4	61.1	64.9	3.1	0.279	0.227	0.797
Ether extract	64.4	65.5	60.5	63.6	2.0	0.085	0.216	0.554

*N* = 40 for total, *n* = 10 for each treatment

^1^The DDGS included 10% corn DDGS in 7 to 11 kg diets and 15% corn DDGS in 11 to 25 kg diets. The SHWB included 6.5% wheat bran and soybean hulls in 7 to 11 kg diets and 6.8% wheat bran and soybean hulls in 11 to 25 kg diets

^2^NSPases containing 0.01% xylanase and 0.05% beta-mannanase was supplemented into experimental diets at 0.06%

### Growth performance

In the present study, BW and ADFI did not differ among any of the treatments (Table 11). Pigs fed SHWB tended to have an increased (*P* = 0.099) ADG during phase 1 (d 0 to 19) but no additional effects were observed during any other period of the study. Additionally, SHWB tended to have higher (*P* = 0.068) G:F during phase 1 and phase 3 (*P* = 0.082), with no effects observed for any other period. Moreover, supplementation of NSPases in DDGS decreased (*P* < 0.05, interaction) G:F during phase 3, however, there was no effect in SHWB.

**Table 11:** Growth performance in pigs fed different NSP sources with or without NSPases

NSP type^1^	DDGS		SHWB		SEM	*P* value		
NSPases^2^	-	+	-	+		NSP	NSPases	NSP × NSPases
BW, kg								
d 0	7.0	6.9	6.9	7.0	0.3	0.944	0.983	0.882
d 19	11.2	11.3	11.9	11.7	0.6	0.366	0.956	0.786
d 37	24.4	24.6	24.7	25.1	1.0	0.686	0.803	0.954
d 61	48.4	48.3	48.7	49.5	1.8	0.665	0.840	0.796
d 86	80.6	81.3	80.0	80.7	2.6	0.727	0.707	0.999
d 103	101.8	102.6	102.3	102.2	4.8	0.972	0.870	0.839
d 120	121.4	122.0	121.7	121.9	6.4	0.978	0.872	0.920
ADG, g/d								
P1 (d 0 to 19)	221	230	259	248	16	0.099	0.936	0.541
P2 (d 19 to 37)	731	736	715	743	30	0.879	0.591	0.700
Nursery overall^3^	469	476	481	489	22	0.587	0.744	0.982
P3 (d 37 to 61)	1,000	987	1,004	1,029	36	0.529	0.872	0.613
P4 (d 61 to 86)	1,395	1,383	1,364	1,362	41	0.509	0.851	0.898
P5 (d 86 to 103)	1,245	1,253	1,313	1,267	144	0.365	0.673	0.550
P6 (d 103 to 120)	1,152	1,147	1,140	1,156	101	0.979	0.924	0.855
Overall^4^	953	959	956	957	51	0.970	0.859	0.900
ADFI, g/d								
P1 (d 0 to 19)	412	408	414	431	13	0.357	0.655	0.441
P2 (d 19 to 37)	1,127	1,138	1,126	1,155	57	0.885	0.726	0.875
Nursery overall	760	763	761	783	33	0.755	0.702	0.771
P3 (d 37 to 61)	1,902	1,993	1,924	1,947	75	0.873	0.447	0.653
P4 (d 61 to 86)	2,898	2,971	2,982	2,944	214	0.699	0.819	0.461
P5 (d 86 to 103)	3,544	3,638	3,644	3,520	394	0.919	0.874	0.291
P6 (d 103 to 120)	3,414	3,482	3,572	3,496	371	0.321	0.963	0.405
Overall	2,204	2,260	2,262	2,233	159	0.757	0.801	0.422
G:F								
P1 (d 0 to 19)	0.53	0.56	0.62	0.57	0.03	0.068	0.581	0.165
P2 (d 19 to 37)	0.65	0.65	0.64	0.65	0.01	0.367	0.670	0.623
Nursery overall	0.62	0.62	0.63	0.62	0.01	0.276	0.930	0.340
P3 (d 37 to 61)	0.53^a^	0.50^b^	0.53^a^	0.53^a^	0.01	0.082	0.106	0.037
P4 (d 61 to 86)	0.45	0.45	0.42	0.43	0.03	0.142	0.946	0.892
P5 (d 86 to 103)	0.35	0.35	0.36	0.36	0.01	0.130	0.576	0.675
P6 (d 103 to 120)	0.34	0.33	0.32	0.33	0.02	0.679	0.975	0.633
Overall	0.43	0.43	0.43	0.43	0.01	0.704	0.704	0.259

^1^The DDGS included 10% corn DDGS in 7 to 11 kg diets and 15% corn DDGS in 11 to 25 kg diets. The SHWB included 6.5% wheat bran and soybean hulls in 7 to 11 kg diets and 6.8% wheat bran and soybean hulls in 11 to 25 kg diets

^2^NSPases containing 0.01% xylanase and 0.05% beta-mannanase was supplemented into experimental diets at 0.06%

^3^Nursery overall, d 0 to 37

^4^Overall, d 0 to 120

^a,b^Within a row, means lacking a common superscript letter differ (*P* < 0.05)

### Carcass traits and uniformity

There were no effects of NSP sources and/or NSPases on loin eye area, hot carcass weight, backfat thickness, loin depth, lean percentage, or dressing percentage at the conclusion of the study (Table 12). Body weight uniformity was not different between the treatments on d 0, 61, 86, or 103 of the study however, pigs in SHWB were more uniform (*P* < 0.05) at d 120 as evidenced by decreased standard deviation and coefficient of variance (Table 13). Additionally, hot carcass weight of pigs tended to be more uniform (*P* = 0.089) in SHWB at the end of the study.

**Table 12:** Carcass traits in finishing pigs fed different NSP sources with or without NSPases

NSP type^1^	DDGS		SHWB		SEM	*P* value		
NSPases^2^	-	+	-	+		NSP	NSPases	NSP × NSPases
LEA^3^, cm^2^	62.71	64.32	63.87	63.48	1.03	0.889	0.563	0.339
HCW^4^, kg	86.4	88.4	87.4	87.4	4.2	1.000	0.645	0.643
BF^5^, mm	16.31	17.07	16.86	17.18	2.49	0.717	0.551	0.804
Loin Depth, mm	69.08	72.96	72.63	71.71	1.51	0.451	0.332	0.120
Lean, %	57.2	57.2	57.3	57.0	1.5	0.915	0.800	0.780
Dressing percentage	72.6	72.5	71.8	71.6	1.1	0.419	0.895	0.997

*N* = 40 for total, *n* = 10 for each treatment

^1^The DDGS included 10% corn DDGS in 7 to 11 kg diets and 15% corn DDGS in 11 to 25 kg diets. The SHWB included 6.5% wheat bran and soybean hulls in 7 to 11 kg diets and 6.8% wheat bran and soybean hulls in 11 to 25 kg diets

^2^NSPases containing 0.01% xylanase and 0.05% beta-mannanase was supplemented into experimental diets at 0.06%

^3^ *LEA* Loin eye area

^4^ *HCW* Hot carcass weight

^5^ *BF* Backfat depth

**Table 13:** Body weight and hot carcass weight uniformity^1^ in pigs fed different NSP sources

NSP type^2^	DDGS	SHWB	DDGS	SHWB	DDGS	SHWB	*P* value (SD)
Body weight	kg	kg	SD^3^	SD	CV^4^	CV	
BW0	7.0	7.0	0.8	0.8	11.93	11.65	0.902
BW61	48.9	49.2	6.7	6.1	13.80	12.36	0.392
BW86	81.0	80.7	9.1	7.7	11.22	9.59	0.264
BW103	102.3	102.9	11.5	9.5	11.19	9.25	0.174
BW120	121.7	121.7	14.1	10.6	11.56	8.68	0.034
HCW	87.4	87.4	8.8	5.6	19.28	12.26	0.089

*N* = 40 for total, *n* = 10 for each treatment

^1^Homogeneity of variance of the residuals was tested using Levene’s test of the GLM procedure, which revealed that the variance of residuals of several dependent variables was unequal. Variances were considered different at *P* ≤ 0.05

^2^The DDGS included 10% corn DDGS in 7 to 11 kg diets and 15% corn DDGS in 11 to 25 kg diets. The SHWB included 6.5% wheat bran and soybean hulls in 7 to 11 kg diets and 6.8% wheat bran and soybean hulls in 11 to 25 kg diets

^3^ *SD* Standard deviation

^4^ *CV* Coefficient of variation

## Discussion

Dietary fiber consists of a wide range of carbohydrates known as NSP that includes hemicelluloses, cellulose, starch, pectins, β-glucan, fructans, and oligosaccharides that are resistant to hydrolysis or cannot be hydrolyzed in the small intestine [42]. The traditional view is that fiber co-products have low nutritional value due to the lower digestible energy and amino acid levels compared to other feedstuffs with relatively higher starch and protein content [43]. High NSP feedstuffs have been limited in nursery diets due to anti-nutritive effects such as reducing nutrient digestibility [6, 44, 45], altering digesta viscosity [46, 47], increasing the proliferation of potentially pathogenic microorganisms [48, 49], and increasing or decreasing the retention time and passage rate of digesta depending on the fiber type [50, 51]. In response, NSPases such as xylanase and mannanase have been developed to hydrolyze NSP present in commonly used feedstuffs and have been shown to increase nutrient digestibility [28, 52], beneficially modulate intestinal health by improving intestinal immune status [21], reduce digesta viscosity [20, 28], and positively impact the relative abundance and diversity of intestinal microbiota [21, 49, 53]. Intestinal microbiota can ferment fiber for their own survival and proliferation and produce gas and organic acids such as short-chain fatty acids and lactic acid, compounds linked with host health and metabolism [54]. The production of organic acids lowers the pH of the intestinal lumen and inhibits the proliferation pathogenic bacteria [54].

In this study, different NSP sources in diets fed to nursery pigs had no effect on Chao-1, Shannon, or Simpson α-diversity at the species level, but supplementation of NSPases increased the Shannon index value. The Shannon index is a well-known diversity index commonly used in microecology studies with higher Shannon index values equating to higher community diversity [55]. The increase in diversity by NSPases in this study may be due to increased amounts of fermented metabolites from fiber hydrolysis [56]. In addition, Quan et al. [57] suggested that a higher Shannon index may be associated with higher feed efficiency, however, the results of the present study do not fully support this, as there were no differences in feed efficiency at the end of phase 2 when microbiota samples were obtained. In the present study, Firmicutes were the predominant phylum observed in the jejunal mucosa of nursery pigs among all treatments, which agrees with the previous study [58]. The SHWB decreased the relative abundance of Proteobacteria, Helicobacteraceae, and *Helicobacter* in the jejunal mucosa of nursery pigs of the present study. Proteobacteria contains many potentially pathogenic microbes such as *Escherichia*, *Campylobacter*, *Salmonella*, *Vibrio*, and *Helicobacter*, and its increase could be considered as a potential indicator of gut dysbiosis [59]. Additionally, SHWB increased the relative abundance of Actinobacteria, which have been shown to produce key antibiotics, immunomodulatory compounds, and metabolites important for host health and homeostasis [60, 61]. Moreover, SHWB increased the relative abundance of Bifidobacterium, a genera reported to enhance gut health and immunity in weaned pigs [62], and reduce pathogen loads post Salmonella challenge [63]. Supplementation of NSPases decreased the relative abundance of Tenericutes and increased Bacteroidetes in the jejunal mucosa. Bacteria in the phylum Tenericutes are characterized as lacking a peptidoglycan cell wall and are generally reported as commensals or obligate parasites of domestic animals [64]. Bacteroidetes are polysaccharide-degrading Gram-negative bacteria that can contribute to the release of energy from fiber and starch [65]. Interestingly, NSPases increased the relative abundance of *Streptococcus*, a genus included in lactic acid bacteria (LAB). The LAB are Gram-positive, catalase-negative rods or cocci that produce lactic acids as their main fermentation product and use carbohydrates as their only or main carbon source [66]. Over 60 genera comprise LAB including *Lactobacillus*, *Weissella*, and *Streptococcus* [67], with *Lactobacillus*, *Enterococcus,* and *Streptococcus* being generally regarded as probiotics in the intestine [68]. Additionally, NSPases increased the relative abundance of *Lactobacillus delbrueckii* in the jejunal mucosa, a species reported to elicit anti-bacterial and anti-adherence effects on *E. coli* [69], *Helicobacter pylori* [70], and *Clostridium difficile* [71]. The gut microbiota is a dynamic community that not only influences the composition of the intestinal mucosa, but also digestion and absorption processes, the production of important metabolites that can play a role in immune development, intestinal morphology, and regulation of host gene expression [72–74].

Nursery pigs fed SHWB had increased concentrations of IgG in the jejunal mucosa. Host defense against infection at mucosal surfaces depends on humoral immunity [75] and IgG contributes to this [76]. Studies investigating the mode of action and role of intestinal IgG in pigs are lacking; however, IgG is hypothesized to play essential roles in the intestinal mucosa through immune cell education, commensal regulation, and systemic immune protection [77]. Moreover, selective symbiotic bacteria have been shown to induce an IgG response, which primary targeted Gram-negative bacterial antigens and conferred protection against systemic infections by *E. coli* and *Salmonella* through opsonization to promote killing by phagocytes [78].

Non-starch polysaccharides are partially fermented by intestinal microbiota resulting in increased short-chain fatty acid production, thus promoting the proliferation of the mucosal epithelium and villus height [79]. Villi in the small intestine are involved mainly in nutrient absorption, thus longer villi can directly affect the nutrient absorption capability in the intestine as it increases the absorptive and surface area [80, 81]. In the present study, SHWB during the nursery phase tended to increase the villus height in the jejunum and tended to decrease the digestibility of ether extract (EE). The reduced digestibility of fat could be explained by increased digesta viscosity [82]. Indeed, jejunal digesta viscosity in the current study was not significantly increased by NSP sources however, the digesta viscosity was numerically higher among both SHWB treatments compared to the DDGS treatment which may have played a role in the tendency to decrease EE digestibility in the present study. Additionally, NSPases tended to increase the AID of DM in this study. Improvements in the digestibility of nutrients with NSPases are more common than improvements in growth in pigs [83] and the results in the present study agree with Passos et al. [28], Casas et al. [84], and Chen et al. [85], that NSPases improve the digestibility of DM in nursery pigs. In the present study, SHWB did not affect growth performance during the overall nursery period (d 0 to 37) but tended to increase ADG and G:F during phase 1 (d 0 to 19) compared to DDGS treatment. These data indicate that nursery pigs may be affected differently by different sources of NSP in nursery diets and NSPases could mitigate the negative effects caused by dietary fiber content.

From phase 3 to phase 6 (d 37 to 120), all pigs were fed a common diet to evaluate the subsequent effects of different feeding strategies in the nursery on growth performance and carcass traits at harvest. Pigs fed SHWB tended to have increased G:F during phase 3, which maybe a result of improved intestinal morphology as seen with the increase in villus height and improving the absorptive surface area for nutrients. Another possible mechanism could be the increased exposure to certain type of NSP in the nursery period and the subsequent shift of the microbiota to a more fiber-degrading composition. Soybean hulls or wheat bran in pig diets have been shown to increase *Lactobacillus* and *Bifidobacteria* among different sections of the intestinal tract [86–89], which may indicate a more primed microbial composition for fiber degradation and utilization. The SHWB in current study had increased relative abundance of *Bifidobacterium* and tended to increase *Lactobacillus* in the mucosa of the jejunum prior to the transition to a common diet. From phase 4 to phase 6 (d 61 to 120), no effects on growth performance were observed among treatments. This lack of a carryover effect on growth performance in the grow-finish phase from pigs that received different diets in the nursery period is not uncommon [90–92] and conflicting results from studies are most likely dependent on several factors such as health status, weaning age, and weaning weight [93–95].

On d 120, all pigs were harvested to obtain carcass data. No differences were observed for any of the treatments on loin eye area, hot carcass weight (HCW), backfat depth, loin depth, lean percentage, and dressing percentage. Interestingly, SHWB decreased the standard deviation for body weight at d 120 and tended to decrease the HCW at harvest. Improved uniformity at the packing plant can have immense implications as variations in carcass size affect the uniformity in meat products and increases the difficulty of handling the carcass and products [96]. Moreover, increased variation at harvest implies there could be relatively more pigs receiving a discount for being under or overweight, thus negatively affecting profitability. In the present study, a total of 15 pigs received a discount at harvest for HCW, nine of which belonged to DDGS treatment. The improved uniformity may be contributed to an increased relative abundance of bacteria associated with fiber degradation and utilization during the nursery phase. This enhancement likely improves the ability of pigs to digest and absorb nutrients more efficiently when feeding a higher fiber diet during growing-finishing phase.

## Conclusion

Soybean hulls and wheat bran replacing DDGS in nursery diets improved uniformity during subsequent production phases in pigs, which might be contributed by improved villi structure and alterations to the intestinal microbiota. Supplementation of NSPases during the nursery phase had beneficial effects on the diversity and composition of the mucosa-associated microbiota, digestibility, and immune status in SHWB treatment, however, had no effects on grow-finish performance or carcass traits.

## Data Availability

All data generated or analyzed during this study are available from the corresponding author upon reasonable request.

## Abbreviations

AA: Amino acid
ADFI: Average daily feed intake
ADG: Average daily gain
AID: Apparent ileal digestibility
BW: Body weight
CP: Crude protein
DDGS: Distillers dried grains with solubles
DM: Dry matter
DNA: Deoxyribonucleic acid
ELISA: Enzyme-linked immunosorbent assay
GE: Gross energy
G:F: Gain to feed ratio
MDA: Malondialdehyde
IL: Interleukin
IgA: Immunoglobulin A
IgG: Immunoglobulin G
PBS: Phosphate-buffered saline
SDF: Soluble dietary fiber
SID: Standard ileal digestibility
STTD: Standardized total tract digestible
TDF: Total dietary fiber
TI: Trypsin inhibitor
TiO_2_: Titanium dioxide
TNF-α: Tumor necrosis factor-alpha
VH:CD: Villus height to crypt depth ratio
